# Energy Requirements of US Army Special Operation Forces During Military Training

**DOI:** 10.3390/nu6051945

**Published:** 2014-05-12

**Authors:** Lee M. Margolis, Aaron P. Crombie, Holly L. McClung, Susan M. McGraw, Jennifer C. Rood, Scott J. Montain, Andrew J. Young

**Affiliations:** 1 Military Nutrition Division, United States Army Research Institute of Environmental Medicine, 15 Kansas Street, Building 42, Natick, MA 01760, USA; E-Mails: aaron.p.crombie.mil@mail.mil (A.P.C.); holly.l.mcclung.civ@mail.mil (H.L.M.); susan.m.mcgraw6.civ@mail.mil (S.M.M.); scott.j.montain.civ@mail.mil (S.J.M.); andrew.j.young.ctr@mail.mil (A.J.Y.); 2 Pennington Biomedical Research Center, Louisiana State University System, Baton Rouge, LA 70808, USA; E-Mail: jennifer.rood@pbrc.edu

**Keywords:** military training, doubly labeled water, energy requirements

## Abstract

Special Operations Forces (SOF) regularly engage in physically demanding combat operations and field training exercises, resulting in high daily energy expenditure, and thus increased energy requirements. However, the majority of studies assessing energy requirements of SOF have been conducted on soldiers going through intense SOF initiation training. The objective of the current investigation was to determine the energy expenditure of SOF conducting military training operations. Thirty-one soldiers taking part in Pre-Mission Training (PMT *n* = 15) and Combat Diver Qualification Courses (CDQC *n* = 16) volunteered to participate in this observational study. Energy expenditure was determined using doubly labeled water. Body weight (83 ± 7 kg) remained stable during both training periods. Overall energy expenditure adjusted for body composition was 17,606 ± 2326 kJ·day^−1^. Energy expenditure was 19,110 ± 1468 kJ·day^−1^ during CDQC and 16,334 ± 2180 kJ·day^−1^ during PMT, with physical activity levels of 2.6 ± 0.2 and 2.2 ± 0.3 during CDQC and PMT, respectively. Compared to the Military Dietary Reference Intakes for energy (13,598 kJ·day^−1^), these data are in agreement with previous reports that energy requirement for SOF Soldiers exceed that of the average soldier.

## 1. Introduction

Similar to athletes, determining energy needs for military personnel enables development of appropriate nutritional strategies that support energy demands during combat operations and field training [1]. Energy requirements for Special Operations Forces (SOF) have been reported to be higher than those necessary to maintain energy balance in average soldiers due to the unique and physically demanding nature of SOF missions [2,3]. Previous investigations have reported that energy expenditures for this unique subset of the military population ranges from ~17,150 kJ·day^−1^ during US Army Ranger School [4] to ~21,750 kJ·day^−1^ during US Army Special Forces Assessment and Selection [5]. The high energy expenditures associated with SOF training result from elevated physical activity levels, a factor determined by total daily energy expenditure over resting metabolic rate. Observed level of physical activity during SOF training has been reported to exceed the upper sustainable limit (>2.5) [6], which if not met with appropriate nutrition intervention will result in weight loss and potentially diminished physical performance [7]. Thus, it has been recommended that dietary energy intakes for SOF exceed 125% of energy needs (13,598 kJ·day^−1^) for the average male soldier established by the Military Dietary Reference Intake (MDRI), which are nutrition guidelines based on the U.S. DRI for all healthy service members age 17–50, for planning, assessment, and development of rations and feeding regimens for the military population [8].

However, most previously reported physical activity levels and energy expenditures for SOF were based on observations of soldiers undergoing intense initial entry training courses, where candidates vie to join the SOF community [4,5,6,9,10]. These rigorous courses are physically demanding, include periods of food restriction, sleep deprivation, and exposure to environmental extremes intentionally designed to push participating soldiers to their maximum limits, to ensure that only the elite complete the training and advance. It has been suggested that dietary recommendations for SOF based on information obtained from these initial training courses may be inflated, and that actual energy needs of SOF personnel engaged in military operations and training are lower than previously suggested. Only a limited number of investigations have been conducted on SOF during garrison activities, the last of which being over a decade old [3]. Furthermore, as athletes’ energy needs fluctuate based on the training cycle [11], likely so do SOF, as these soldiers are continuously participating in intense specialized training programs in preparation for their next deployment. Therefore, assessment of the energy expenditure of qualified SOF during various training activities was warranted, as this knowledge has important policy implications which may influence budget and allocation of foods.

Two garrison-based training programs believed to span the intensity of SOF training were selected for observation. Pre-Mission Training (PMT), where participating soldiers conduct routine training operations before combat deployment and Combat Diver Qualification Course (CDQC), a rigorous, select training program. The objective of the present investigation was to characterize energy requirements of SOF soldiers through the measurement of total daily energy expenditure and physical activity level during PMT and CDQC and compare levels of energy expenditure to the MDRI. We hypothesized that both courses would elicit energy expenditures exceeding the MDRI for energy and that physical activity level would be above the upper sustainable limit (>2.5).

## 2. Experimental Section

### 2.1. Experimental Design

**Figure 1:**
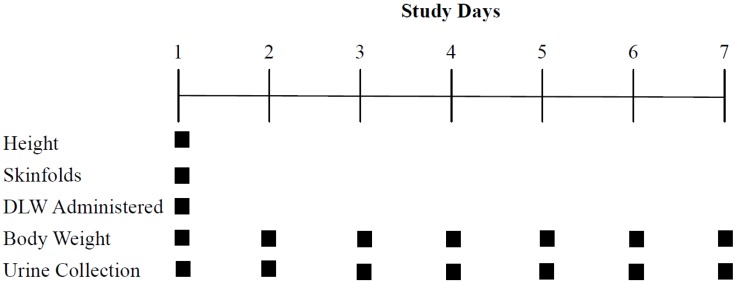
Experimental design. DLW: doubly labeled water.

Thirty-one male soldiers participating in CDQC and PMT volunteered to participate in this 7-day observational study. Two volunteers were medically withdrawn from CDQC during data collection; as such, data were collected and analyzed on 29 soldiers (CDQC; *n* = 14, PMT; *n* = 15). The experimental design is depicted in Figure 1. Energy expenditure and body weight were assessed daily each observation period. Participation in the study was voluntary, with written consent being obtained from each soldier before the initiation of data collection. This study was conducted after review and approval by the US Army Research Institute of Environmental Medicine Human Use Review Committee. Investigators adhered to US Army Regulation 70-25 and US Army Medical Research and Material Command regulation 70-25 on the participation of volunteers in research.

### 2.2. Training Description

During PMT, soldiers practice skills required to conduct SOF combat operations. The study participants completed physical training, weapons familiarization, airborne operations, urban operations, and convoy operations. The Combat Diver Qualification Course is a highly specialized course teaching soldiers how to conduct underwater military operations. During the observation period, participants engaged in daily physical training, that included formation runs up to five miles, rigorous callisthenic-type workout, 4 h of high-intensity pool work, open water swims, and drills to properly don 30 kg open circuit diving gear.

### 2.3. Anthropometrics

Height, body weight, and body composition were assessed on day 1 of data collection. Vertical height was measured using a stadiometer (Seca; Creative Health Products, Plymouth, MI, USA) to the nearest 0.1 cm. Body weight was measured using an electronic scale (Befour model PS6600; Befour Inc., Saukville, WI, USA) calibrated to industry standards using standard weights accurate to 0.1 kg. Body composition was determined from skinfold thickness measurements using Lange calipers (Beta Technology, Santa Cruz, CA, USA) made at 4 skinfold sites (biceps, triceps, subscapular, suprailiac) [12]. Participant’s weight was measured each subsequent day of data collection.

### 2.4. Total Daily Energy Expenditure

Total daily energy expenditure was assessed using doubly labeled water (DLW). This method has previously been validated for a military population and details of the procedure have been reported [13]. Briefly, participants fasted for 4-h prior to ingestion of DLW on day 1 to stabilize total body water, and to minimize the likelihood of the isotope binding to digestible carbohydrate. Before consuming DLW, a baseline urine sample was collected to determine background abundance of ^18^O and ^2^H. To minimize disruption in the participants training an overnight dosing protocol was utilized. Following the ingestion of the DLW (0.23 g H_2_^18^O·kg·TBW^−1^ and 0.15 g ^2^H_2_O·kg·TBW^−1^; Sigma-Aldrich, St. Louis, MO, USA) participants slept, remaining in a fasted state for 8-h. Upon waking participants provided first and second-morning void urine samples on day 2 of the study. Total body water was calculated by determining the regression line for the elimination of ^2^H and ^18^O and extrapolated to a maximum enrichment.

Second-morning void urine samples were collected each morning during the 6-day observation period. Enrichments of ^2^H and ^18^O were measured using isotope ratio mass spectroscopy (Finnigan Mat 252, Thermo Fisher Scientific, Waltham, MA, USA). The ^2^H and ^18^O isotope elimination rates (k_H_ and k_O_) were calculated by linear regression using the isotopic disappearance rates in the urine samples collected during the 6-day study to determine CO_2_ production according to Schoeller *et al.* [14]:

rCO_2_ (moL∙day^−1^) = (*N*/2.078) (1.01 k_O_ − 1.04 k_H_) − 0.0246 rH_2_O_f_ (1)

where *N* is total body water; k_O_ and k_H_ are ^18^O and ^2^H isotope disappearance rates, respectively; and rH2Of is the rate of fractionated evaporated water loss and is estimated to be 1.05 *N* × (1.01 k_O_ − 1.04 k_H_). Energy expenditure was calculated using the energy equivalent of CO_2_ for a respiratory quotient of 0.86 based on average food quotient for the course [15].

To account for the natural abundance of ^2^H and ^18^O in local drinking water, 2 participants from each training course were randomly chosen to consume local drinking water rather than DLW to serve as a control group. Additionally, local water was analyzed independently to determine ^2^H and ^18^O enrichments.

Resting metabolic rate (RMR) was estimated using measures of fat-free mass (FFM) with the following equation [16]:

RMR (kJ∙day^−1^) = 370 + (21.6 × FFM) × 4.184 (2)

Diet-induced thermogenesis was calculated as 10% energy expenditure [17]. Activity-induced energy expenditure was derived from total daily energy expenditure minus resting metabolic rate and diet-induce thermogenesis [7]. Physical activity level was defined as a ratio between energy expenditure and calculated resting metabolic rate [18].

### 2.5. Statistical Analysis

Common descriptive statistics were used for baseline characteristics total daily energy expenditure, activity-induced energy expenditure, RMR, and physical activity level. Independent *t*-tests were utilized to compare characteristics. Change in body weight from day 1 to 7 was assessed using a paired *t*-test. A one samples *t*-test was used to compare energy expenditures of the current investigation to the MDRI for energy needs of the average male soldier. An analysis of covariance, with fat mass and fat-free mass as covariates, was conducted to assess if range of energy expenditure in PMT and CDQC was due to difference in groups body composition. A regression model adjusting for fat mass and fat-free mass was utilized to adjust average energy expenditure between trainings. The alpha level for significance was set at *P* < 0.05 and data are presented as mean ± SD. Statistical analysis was conducted using the SPSS statistical package version 20.0 (SPSS Inc., Chicago, IL, USA).

## 3. Results

### 3.1. Participant Characteristics

Overall, participating soldiers had been a part of a SOF unit for 3 ± 3 years. Participant characteristics are shown in Table 1. Though there was no difference in initial body weight (*P* > 0.05), Soldiers participating in PMT had higher (*P* < 0.05) percent body fat and fat mass, with no difference in fat-free mass (*P* > 0.05). Body weight was maintained (83 ± 7 kg, *P* > 0.05) during PMT and CDQC.

**Table 1:** Participant characteristics.

Characteristics	CDQC (*n* = 14)	PMT (*n* = 15)
Age (years)	28 ± 4	30 ± 7
Weight (kg)	82 ± 7	84 ± 7
Height (cm)	181 ± 5	177 ± 5 *
BMI (kg/m^2^)	22 ± 5	25 ± 4
Percent Body Fat (%)	14 ± 3	18 ± 4 *
Fat Mass (kg)	12 ± 3	16 ± 4 *
Fat-Free Mass (kg)	69 ± 5	70 ± 4

Values are mean ± SD. * Different from Combat Diver Qualification Course, *P* < 0.05.

### 3.2. Total Daily Energy Expenditure

**Figure 2:**
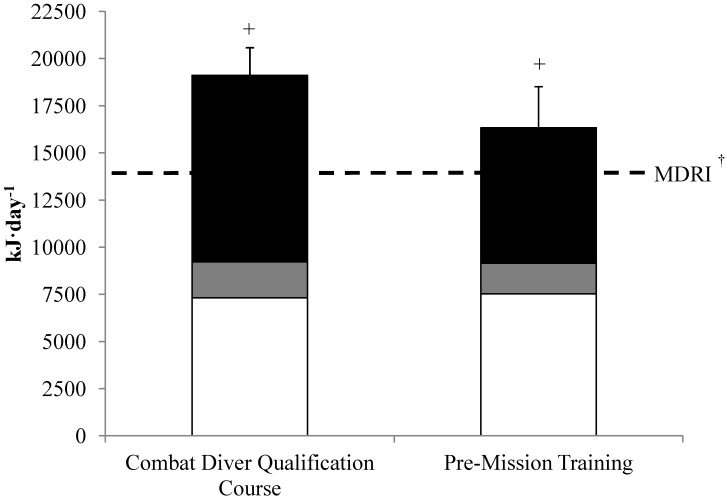
Total daily energy expenditure. Values are mean ± SD. (□) resting metabolic rate, (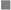) diet-induced thermogenesis, (■) activity-induced energy expenditure. ^+^ Total daily energy expenditure different from MDRI, *P* < 0.05; ^†^ MDRI; military dietary reference intakes 13,598 kJ·day^−1^.

Compared to the MDRI for energy both CDQC and PMT elicited energy expenditure exceeding (*P* < 0.05) energy needs (13,598 kJ·day^−1^) for the average male soldier. Overall, adjusted total daily energy expenditure for the two training periods was 17,606 ± 2326 kJ·day^−1^. Background enrichments of ^2^H and ^18^O for placebo control participants during PMT and CDQC remained constant throughout the training period. Energy expenditure during CDQC was 19,110 ± 1468 kJ·day^−1^, while observed energy expenditure during PMT was of 16,334 ± 2180 kJ·day^−1^ (Figure 2). This range in energy expenditure between PMT and CDQC was maintained after adjusting for fat mass and fat-free mass as covariates. Resting metabolic rate was 7313 ± 473 kJ·day^−1^ for soldiers taking part in CDQC and 7524 ± 420 kJ·day^−1^ for soldiers conducting PMT. Physical activity levels were 2.6 ± 0.2 and 2.2 ± 0.3 during CDQC and PMT, respectively, with activity-induced energy expenditure accounting for 52% (9886 ± 1068 kJ·day^−1^) of total daily energy expenditure during CDQC and 44% (7177 ± 1843 kJ·day^−1^) of total daily energy expenditure during PMT.

## 4. Discussion

The major finding of the present observational investigation was that total daily energy expenditure of SOF during PMT and CDQC was 120% and 140%, respectively, higher than energy requirements set by the MDRI (13,598 kJ·day^−1^). Additionally, the exceedingly high levels of total daily energy expenditure observed during the two distinct training periods appears to be driven by activity induced energy expenditures due to high physical activity levels. This is an important observation when considering feeding regimens and nutrition policies for SOF to ensure they receive adequate daily energy to support their intense workload.

Findings in the present study are similar to previously reported energy expenditures of SOF conducting military operations [3,19,20]. Participants completing standard military tasks during PMT experienced energy expenditures of 16,334 ± 2180 kJ·day^−1^, which are comparable to those reported by Tharion *et al.* [3], who stated that energy needs of SOF conducting nine days of routine garrison training, consisting of rock climbing, simulated urban combat, and weapons familiarization were 17,150 ± 3096 kJ·day^−1^. Energy expenditure during CDQC exceeded those observed during PMT. Differences in energy expenditure between the two training periods may be explained by differences in activity-induced energy expenditure due to a higher physical activity level during CDQC. The higher energy expenditure observed during CDQC (19,110 ± 1468 kJ·day^−1^) is similar to reported energy expenditures (19,071 ± 2368 kJ·day^−1^) of SOF conducting high altitude military operations [19]. In the current investigation, discrepancies in body composition between training groups does not appear to account for differences in the span of energy expenditure between PMT and CDQC, as fat mass and fat-free mass were adjusted for as covariates. Differences in fat mass may be attributed to the fact that CDQC is one of the more rigorous training programs that SOF will undertake, which most participating soldiers will specifically train-up for. As such, these may account for subjects participating in CDQC being leaner.

The range of energy expenditures observed in qualified SOF are comparable to those reported during initial SOF training courses. In previous investigations conducted during US Army Ranger School and Special Forces Qualification Course, it has been reported that participating soldiers expended 17,113 ± 2000 kJ·day^−1^ and 18,832 ± 4155 kJ·day^−1^, respectively [4,5,6]. Though initial training to become a member of the SOF community is designed to be intentionally strenuous, these findings show that the rigors of SOF training to maintain military proficiency in preparation for unique and physically demanding missions elicit similar energy expenditures. Foremost, this suggests that previous dietary recommendations for additional energy were appropriate for this unique subset of the military population. Additionally, these findings indicate that SOF initial training courses are appropriate to use as a model to assess physiological strain of military operations in a controlled environment.

Black *et al.* [21] hypothesized that a range exists for sustainable physical activity levels, with the upper limit being from 2.2 to 2.5. Levels beyond 2.5 are believed to be difficult to maintain over long periods of time [7], resulting in reductions in body weight as energy intake may not be adequately increased to compensate for large and prolonged elevations in energy expenditure, leading to a negative energy balance [22]. However, it is not uncommon for athletes [23,24,25], first responders [26], and members of the military [6,27] to experience high energy expenditures and physical activity levels which exceed 2.5. Wildfire suppression, which can last numerous days and requires firefighters to perform under demanding conditions, has been reported to elicit energy expenditures of 20,401 ± 3000 kJ·day^−1^ with physical activity levels reaching 2.8 ± 0.5 for five days [26]. Westerterp *et al.* [25] reported that during the Tour de France, a cyclist maintains a physical activity level of ~4.0, with a peak value of 5.2 and mean daily energy expenditures of 35,677 kJ·day^−1^ for several weeks. In both the wildfire suppression and Tour de France study, body weight was maintained. While physical activity levels in the current study (PMT; 2.2 ± 0.3 and CDQC; 2.6 ± 0.2) did not reach levels of these previous investigations, they were at or above the upper limit with no alteration in body weight. Had the present observation period been longer than seven days, weight loss may have occurred, as these levels of intense physical activity may become too great to sustain. However, for the short-term, these data suggest that if appropriate accommodations are made to energy intake, physical activity levels beyond 2.5 can be sustained with no detriment to body weight. Important to note that in the present study, fat-free mass which was used to calculate RMR and thus physical activity level was determined using skin-fold measurements, a field expedient method to measure body composition. This method is less accurate than the gold standard dual energy X-ray absorptiometry (DXA) to estimate body composition. Inaccuracies associated with skinfold methods may have resulted in an underestimation of fat-free mass and RMR and over inflated physical activity level. This likely is not the case in the present investigation, however, fat-free mass in our participants is greater than that reported in previous studies in the SOF population [3,10,19]. Regardless, future investigations conducted with the SOF community should consider using more accurate methods to assess body composition (DXA scan) and resting metabolic rate (metabolic cart).

Inadequate energy intakes during periods of high metabolic demand are common when soldiers are conducting intense field training exercises, and dependent on combat rations as their only source of food [28]. Energy deficits may reach roughly 40% of total energy needs [29]. In the present study, despite high energy expenditures due to elevated physical activity, there was no reduction in body weight following the seven-day training period. Though it is a limitation to this study that energy intake was not assessed to determine energy balance, the maintenance of body weight suggests that energy requirements were met by dietary intake. This may be due to the fact that all meals during the observation period were consumed in a military dining facility (ad libitum cafeteria-style dining) and that participants in CDQC were provided with ad libitum liquid carbohydrate supplements. This would be consistent with historical reports that for a military population, energy balance is easier to maintain when hot meals are provided in a dining facility *versus* combat rations [28], and more recent findings that providing energy dense dietary supplements can reduce losses in body mass associated with military training [30]. Specific to SOF, a recent report from our laboratory [6] demonstrated that during strenuous SOF training when hot meals are provided ad libitum, energy intake met or exceed energy expenditures, with energy intake being roughly 9205 kJ·day^−1^ higher when soldiers consumed three meals per day in a dining facility compared three combat rations per day during Special Forces Qualification Course. Furthermore, during U.S. Navy Sea, Air, and Land (SEAL) Hell Week, which is considered by many to be one of the most arduous military training periods, positive energy balance was achieved, consuming 24,401 ± 4799 kJ·day^−1^ when provided four hot meals per day with an energy expenditure of 22,443 ± 2309 kJ·day^−1^ [31]. While it appears that energy balance may be maintained when soldiers are provided hot meals, this is not always feasible as mission objectives typically curtail time to eat and food availability. For future investigations in field operations, the utilization of energy dense supplements may be a promising countermeasure to prolong sustainment of high energy expenditures and physical activity levels.

## 5. Conclusions

Energy needs of SOF appear to be ~17,600 kJ·day^−1^, with differences in physical activity levels (2.2–2.6) during specific training periods eliciting variations in energy expenditure ranging from 16,700 to 19,200 kJ·day^−1^. Findings from the current investigation suggest that dietary energy requirements of SOF Soldiers exceed 120%–140% of the MDRI. Additionally, the level of energy expenditures observed during PMT and CDQC are similar to those observed during SOF initial entry training, suggesting that though SOF initial entry training is intentionally rigorous, it may be appropriate to use as a model to assess physiological strain of military operations in this unique population in a controlled environment. Determining total daily energy expenditure of SOF is paramount for creation of appropriate nutrition policies and food allocation to support the physically demanding workload of this unique subset of the military population.

## References

[1] Montain S.J., Young A.J. (2003). Diet and physical performance. Appetite.

[2] Tharion W.J., Lieberman H.R., Montain S.J., Young A.J., Baker-Fulco C.J., Delany J.P., Hoyt R.W. (2005). Energy requirements of military personnel. Appetite.

[3] Tharion W.J., Baker-Fulco C.J., Bovill M.E., Montain S.M., DeLany J.P., Champagne C.M., Hoyt R.W., Lieberman H.R. (2004). Adequacy of garrison feeding for special forces soldiers during training. Mil. Med..

[4] Shippee R.L., Friedl K.E., Kramer T., Mays M., Popp K., Askew E.W., Fairbrother B., Hoyt R., Vogel J., Marchitelli L. (1994). Nutritional and Immunological Assessment of Ranger Students with Increased Caloric Intake.

[5] Fairbrother B., Shippee R.L., Kramer T., Askew E.W., Mays M.Z., Popp K., Kramer M., Hoyt R.W., Tulley R., Rood J. (1995). Nutritional and Immunological Assessment of Soldiers during the Special Forces Assessment and Selection Course.

[6] Margolis L.M., Rood J., Champagne C., Young A.J., Castellani J.W. (2013). Energy balance and body composition during us army special forces training. Appl. Physiol. Nutr. Metab..

[7] Westerterp K.R. (2013). Physical activity and physical activity induced energy expenditure in humans: Measurement, determinants, and effects. Front. Physiol..

[8] Army Regulation 40-25. Nutrition Standards and Education. http://www.apd.army.mil/pdffiles/r40_25.pdf.

[9] Nindl B.C., Barnes B.R., Alemany J.A., Frykman P.N., Shippee R.L., Friedl K.E. (2007). Physiological consequences of U.S. Army ranger training. Med. Sci. Sports Exerc..

[10] Friedl K.E., Moore R.J., Hoyt R.W., Marchitelli L.J., Martinez-Lopez L.E., Askew E.W. (2000). Endocrine markers of semistarvation in healthy lean men in a multistressor environment. J. Appl. Physiol..

[11] Loucks A.B., Kiens B., Wright H.H. (2011). Energy availability in athletes. J. Sports Sci..

[12] Durnin J.V., Womersley J. (1974). Body fat assessed from total body density and its estimation from skinfold thickness: Measurements on 481 men and women aged from 16 to 72 years. Br. J. Nutr..

[13] DeLany J.P., Schoeller D.A., Hoyt R.W., Askew E.W., Sharp M.A. (1989). Field use of D2 ^18^O to measure energy expenditure of soldiers at different energy intakes. J. Appl. Physiol..

[14] Schoeller D.A., Ravussin E., Schutz Y., Acheson K.J., Baertschi P., Jequier E. (1986). Energy expenditure by doubly labeled water: Validation in humans and proposed calculation. Am. J. Physiol..

[15] Wolfe R.R. (2005). Isotope Tracers in Metabolic Research: Principles and Practice of Kinetic Analysis.

[16] Cunningham J.J. (1991). Body composition as a determinant of energy expenditure: A synthetic review and a proposed general prediction equation. Am. J. Clin. Nutr..

[17] Westerterp K.R. (2004). Diet induced thermogenesis. Nutr. Metab..

[18] Westerterp K.R. (2001). Limits to sustainable human metabolic rate. J. Exp. Biol..

[19] Hoyt R.W., Jones T.E., Baker-Fulco C.J., Schoeller D.A., Schoene R.B., Schwartz R.S., Askew E.W., Cymerman A. (1994). Doubly labeled water measurement of human energy expenditure during exercise at high altitude. Am. J. Physiol..

[20] Tharion W.J., Warber J.P., Hoyt R.W., DeLany J.P. (1998). Energy requirements of rangers in garrison and in the field. FASEB J..

[21] Black A.E., Coward W.A., Cole T.J., Prentice A.M. (1996). Human energy expenditure in affluent societies: An analysis of 574 doubly-labelled water measurements. Eur. J. Clin. Nutr..

[22] Melzer K., Kayser B., Saris W.H., Pichard C. (2005). Effects of physical activity on food intake. Clin. Nutr..

[23] Hill R.J., Davies P.S. (2001). Energy expenditure during 2 wk of an ultra-endurance run around australia. Med. Sci. Sports Exerc..

[24] Hill R.J., Davies P.S. (2002). Energy intake and energy expenditure in elite lightweight female rowers. Med. Sci. Sports Exerc..

[25] Westerterp K.R., Saris W.H., van Es M., ten Hoor F. (1986). Use of the doubly labeled water technique in humans during heavy sustained exercise. J. Appl. Physiol..

[26] Ruby B.C., Shriver T.C., Zderic T.W., Sharkey B.J., Burks C., Tysk S. (2002). Total energy expenditure during arduous wildfire suppression. Med. Sci. Sports Exerc..

[27] Hoyt R.W., Friedl K.E. (2006). Field studies of exercise and food deprivation. Curr. Opin. Clin. Nutr. Metab. Care.

[28] Marriott B.M. (1995). Not Eating Enough: Overcoming Underconsumption of Military Operational Rations.

[29] Montain S.J. (2006). Physiological demand of combat operations. Nutrient Composition of Rations for Short-Term, High-Intensity Combat Operations.

[30] Diment B.C., Fortes M.B., Greeves J.P., Casey A., Costa R.J., Walters R., Walsh N.P. (2012). Effect of daily mixed nutritional supplementation on immune indices in soldiers undertaking an 8-week arduous training programme. Eur. J. Appl. Physiol..

[31] Seale J.L., Thorp J.W., Conway J.M., Rumpler W.V., Haberman K.J. (1994). Energy expenditure and fluid production in hyperbaric He-O2 environments using doubly labeled water. Undersea Hyperb. Med..

